# Functional characterization of α-Gal producing lactic acid bacteria with potential probiotic properties

**DOI:** 10.1038/s41598-022-11632-8

**Published:** 2022-05-06

**Authors:** Timothy Bamgbose, Pilar Alberdi, Isa O. Abdullahi, Helen I. Inabo, Mohammed Bello, Swati Sinha, Anupkumar R. Anvikar, Lourdes Mateos-Hernandez, Edgar Torres-Maravilla, Luis G. Bermúdez-Humarán, Alejandro Cabezas-Cruz, Jose de la Fuente

**Affiliations:** 1 ICMR-National Institute of Malaria Research, Sector 8, Dwarka, New Delhi, India; 2 Instituto de Investigación en Recursos Cinegéticos IREC-CSIC-UCLM-JCCM, SaBio, Ronda de Toledo s/n, 13005 Ciudad Real, Spain; 3 Department of Microbiology, Ahmadu Bello University, Samaru Zaria, Kaduna, Nigeria; 4 Department of Veterinary Public Health and Preventive Medicine, Ahmadu Bello University, Samaru Zaria, Kaduna, Nigeria; 5 UMR BIPAR, INRAE, ANSES, Ecole Nationale Vétérinaire d’Alfort, Université Paris-Est, 94700 Maisons-Alfort, France; 6 INRAE, AgroParisTech, Micalis Institute, Université Paris-Saclay, 78350 Jouy-en-Josas, France; 7 Department of Veterinary Pathobiology, Center for Veterinary Health Sciences, Oklahoma State University, Stillwater, OK 74078 USA

**Keywords:** Drug discovery, Immunology, Microbiology

## Abstract

The possibility of exploiting the human immune response to glycan α-Gal for the control of multiple infectious diseases has been the objective of recent investigations. In this field of research, the strain of *Escherichia coli* O86:B7 has been at the forefront, but this Gram-negative microorganism presents a safety concern and therefore cannot be considered as a probiotic. To address this challenge, this study explored the identification of novel lactic acid bacteria with a safe history of use, producing α-Gal and having probiotic potential. The lactic acid bacteria were isolated from different traditionally fermented foods (*kununn-zaki*, *kindirmo*, and *pulque*) and were screened for the production of α-Gal and some specific probiotic potential indicators. The results showed that Ten (10) out of forty (40) [25%] of the tested lactic acid bacteria (LAB) produced α-Gal and were identified as *Limosilactobacillus fermentum, Levilactobacillus brevis, Agrilactobacillus composti, Lacticaseibacillus paracasei*, *Leuconostoc mesenteroides* and *Weissella confusa*. Four (4) LAB strains with highest levels of α-Gal were further selected for in vivo study using a mouse model (α1,3GT KO mice) to elucidate the immunological response to α-Gal. The level of anti-α-Gal IgG observed were not significant while the level of anti-α-Gal IgM was lower in comparison to the level elicited by *E*. *coli* O86:B7. We concluded that the lactic acid bacteria in this study producing α-Gal have potential probiotic capacity and can be further explored in α-Gal-focused research for both the prevention and treatment of various infectious diseases and probiotic development.

## Introduction

The burden of various infectious diseases caused by parasitic agents such as *Trypanosoma* spp., *Leishmania* spp., *Plasmodium* spp.^1–3^ and several others tick-borne infectious agents^4^ is enormous. While the ever-evolving system of the parasite-host relationship makes prophylaxis a challenging one and a reason to develop an alternative to antibiotics^5^. Probiotics^6^ and vaccines^7^ represent an interesting alternative in solving the twenty-first century challenge in which α-Gal immunity is receiving much interest as a potential candidate for the development of new probiotic vaccines^8,9^.

According to the Food and Agriculture Organization of the United Nations and the WHO (FAO/WHO) the definition of probiotics centers on the usage of live microorganisms that, when administered in adequate amounts of 10^8^–10^10^ CFU/ml daily, confer a health benefit on the host^10^. While α-Gal is a carbohydrate moiety Galα1-3Galβ1-4GlcNAc-R simply referred to as α-Gal, a glycan produced by some bacteria especially of the Enterobacteriaceae family^11,12^*.* Its expression in Enterobacteriaceae is associated with the bacterial cell wall glycoprotein, capsule and/or lipopolysaccharide (LPS)^13^. Non-primate mammals, New World Monkeys, lemurs, mice, and some parasite such as *Plasmodium* also produce this glycan as they have a functional alpha-1,3-galactosyltransferase gene which is responsible for the production of α-Gal^14,15^.

However, in humans the α-1,3GT gene encoding the enzymes that synthesize the carbohydrate have been inactivated during evolution and do not produce the glycan, resulting in the ability to generate high titers of antibodies against α-Gal^16–18^. Thus, all sources of α-Gal in humans originate elsewhere, to the extent that in healthy humans about 5% of the IgM and IgG present in the blood is directed against the α-Gal antigen. The source of the α-Gal antigen will determine whether the immune response will be beneficial or detrimental; IgG and IgM immune responses are beneficial while IgE antibody triggers anaphylaxis^19^.

The beneficial immune response often is from microbes that produce α-Gal, therefore modulation of the gut microbiome with such microbes might be of advantage. However, most of the reported microbes producing the glycan has a pathogenic strain of the species, notably is *Escherichia coli*^20^, *Aeromonas veronii* and *Pseudomonas entomophila*^21^ thus making their usage for gut modulation comes with safety challenge. Hence, the focus is to investigate whether lactic acid bacteria (LAB) isolated from traditional fermented food can also produce the glycan α-Gal as well as study their ability to stimulate immunological response. Lactic acid bacteria are Gram-positive bacteria that produce no spore, facultative anaerobes, non-flagellate, have a negative reaction to catalase and has lactic acid as a product of their sugar fermentation. According to their glucose fermentation pathway, those that produce lactic acid as the sole product are homofermentative while those that produce other organic acids alongside carbon dioxide are categorized as heterofermentative^22,23^. They include several bacterial genera mainly, of which the most studied are *Lactobacillus*, *Lactococcus*, *Streptococcus*, *Enterococcus*, and *Pediococcus*. They are widely used in industrial food fermentation processes and some genera, such as *Lactobacillus*, are commensal bacteria that are part of the intestinal microbiota and abundant in human vagina. The *Lactobacillus* that was described as *Lactobacillus delbrueckii* in 1901 retained 35 species while the remaining species were placed under *Paralactobacillus* and 23 other genera. The 23 novel genera include *Amylolactobacillus, Acetilactobacillus, Agrilactobacillus, Apilactobacillus, Bombilactobacillus, Companilactobacillus, Dellaglioa, Fructilactobacillus, Furfurilactobacillus, Holzapfelia, Lacticaseibacillus, Lactiplantibacillus, Lapidilactobacillus, Latilactobacillus, Lentilactobacillus, Levilactobacillus, Ligilactobacillus, Limosilactobacillus, Liquorilactobacillus, Loigolactobacilus, Paucilactobacillus, Schleiferilactobacillus, and Secundilactobacillus*^24^. Lactic acid bacteria are generally recognized as catalase negative^24^, safe by health authorities and have a Generally Recognized as Safe (GRAS) status as stipulated by US Food and Drug Administration (FDA, USA) and Qualified Presumption of Safety (QPS) profile by European Food Safety Authority (EFSA; http://www.efsa.europa.eu/en/efsajournal/doc/587.pdf). Lactic acid bacteria are naturally abundant in food^25,26^, well characterized and most studied for probiotic usage among several types of microbes^27,28^ with the *Lactobacillus* genus being the most important in food processing within the group^29,30^.

Isolation, identification and screening of LAB producing α-Gal were carried out with the aim of searching for a new safe alternative microorganism that can be used in both human and animal health applications instead of *E. coli* 086:B7. Yilmaz et al.^20^ had previously reported that *E. coli* 086:B7, a human pathobiont producing α-Gal, elicited an immune response in α-1,3GT-deficient mice that could block the transmission of *Plasmodium* sporozoite, which also produces the same glycan. The study suggests that *E. coli* 086:B7 can be used as a probiotic to prevent malaria transmission. However, since this is a pathogenic strain, it may not be an ideal candidate for probiotic use due to safety concerns.

Furthermore, the isolates from this study, due to their ability to produce α-Gal, can also be considered for the development of an α-Gal glycovaccine or used as a postbiotics by inducing the α-Gal immune response for the prevention of various infectious diseases^9^. This consideration is based on the reports by Mateos-Hernández et al.^31^, in which a potential α-Gal-producing probiotic bacterium protects birds from *Aspergillus fumigatus* infection, as well as studies by Pacheco et al.^2,21^, in which α-Gal-vaccinated zebrafish were protected against *Mycobacterium marinum* infection. The α-Gal-induced immune response has also been proposed for the prevention of malaria and tuberculosis^13,20^.

In the race to win the battle against COVID-19, results have suggested a role for anti-α-Gal immune response in the disease prevention and control^32–34^. Mak et al.^35^ suggested that a probiotic against SARS-COV-2 infection would most likely be a new bacterial strain having a therapeutic potential against Covid-19 for gut modulation or a genetically modified probiotics for mucosal immune stimulation^36^. In addition, Coronaviruses have been reported to contain a glycan shield due to the post-translational modification of their protein envelope that renders the virus susceptible to neutralization by anti-glycan antibodies^37^. This has generated the hypothesis that if the repertoire of anti-α-Gal antibodies can be increased in the human population by exposing it to bacteria producing α-Gal it could be a means to reduce or eliminate transmission of the deadly virus^37^. A hypothesis also proposed by de la Fuente et al.^38^ considers that interaction of arthropods with humans that instigate an immune response to α-Gal may be a means to prevent transmission of SARS-COV-2. The report from this study on α-Gal-producing LAB with probiotic potential provides further paths to be explored.

In the area of studying probiotics for health purposes, several species of *Lactobacillus*, *Bifidobacterium* and *Streptococcus* have been used, while some species of *Enterococcus* and *Lactococcus* have garnered some interest^39–41^. One of these species is *Lactobacillus casei*, a probiotic used for the treatment of diarrhea which also produce α-Gal but does not stimulate anti-α-Gal immune response in humans^42^. However, the genus *Lactobacillus* counts more than two hundred and fifty species and nineteen subspecies described so far^24^. Hence, this diversity makes them good candidates to explore their probiotic potential and their α-Gal producing abillity for the prevention and treatment of different infectious diseases as the health properties are strain-specific^13,17,19^. Thus, this study focused on exploring a potential probiotic candidate strain from fermented food that can produce α-Gal and their in vivo study for eliciting anti-α-Gal IgM and IgG. The experimental objective of the study was to find other bacteria aside from *E. coli* with no safety issues that produce α-Gal and further investigate if it can bring about an immunological response in the murine model.

## Results

### Lactic acid bacteria produce α-Gal

**Figure 1:**
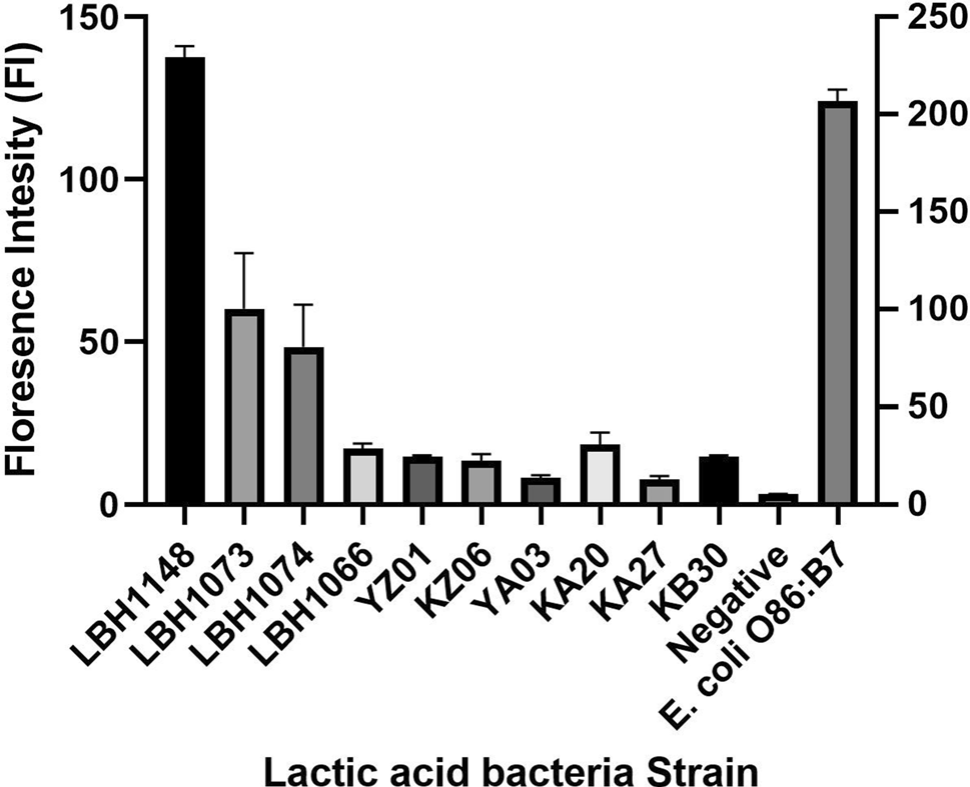
The α-Gal level of selected lactic acid bacteria. The α-Gal levels in lactic acid bacteria isolated from traditionally fermented food is presented as the geo mean of the florescence intensity. Bacterial isolates are represented in bar chart to show the fluorescence intensity as against the negative control (bacteria with secondary antibody only) and *E. coli* O86:B7 served as the positive control. The LBH1148, LBH1073, LBH1074, LBH1066, YZ01, KZ06, YA03, KA20, KA27, KB30 represent lactic acid bacteria strains that were further identified molecularly.

Out of the 40 LAB isolated from *kununn-zaki*, *kindirmo*, and *pulque*, 10 were positive for α-Gal with the isolates having a varying concentration of produced α-Gal (Fig. 1).

### Taxonomic classification of α-Gal producing lactic acid bacteria

**Figure 2:**
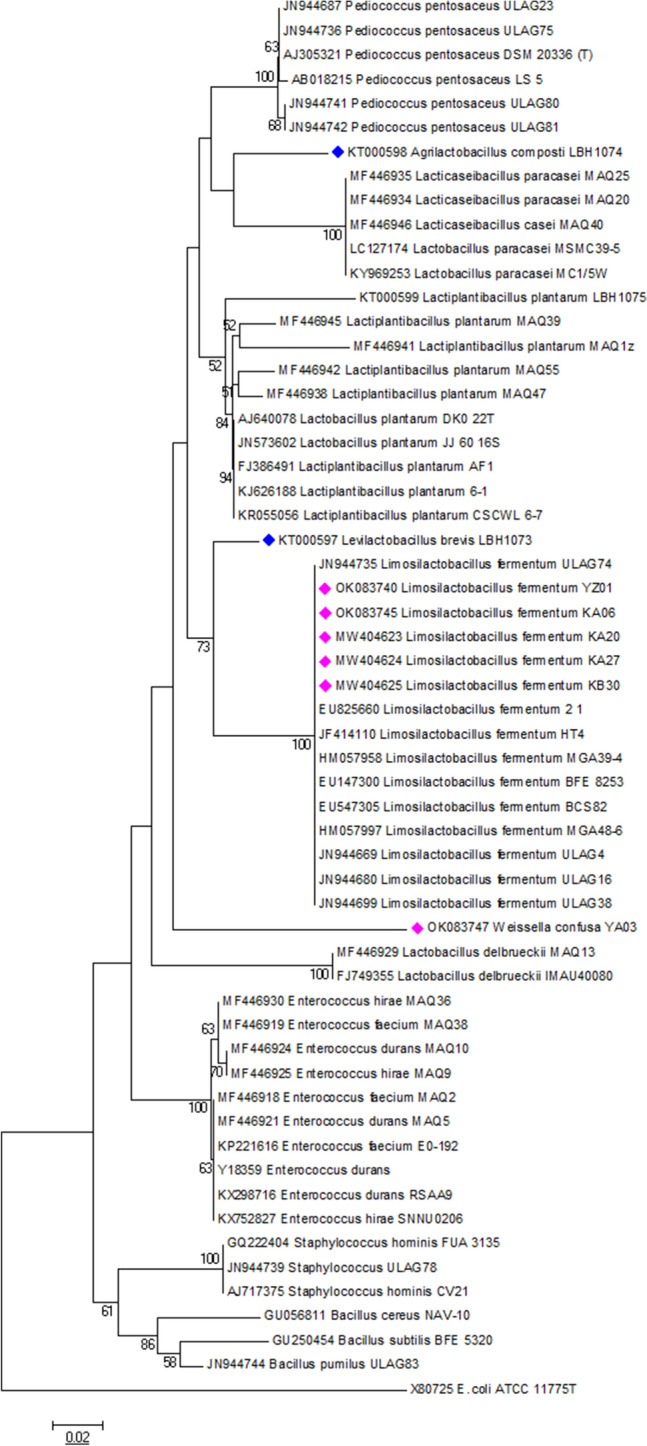
Phylogenetic tree of lactic acid bacteria producing α-Gal based on the alignment of the partial 16S rRNA sequences of the LAB isolates from this study. The evolutionary history was inferred using the Neighbor-Joining method. The optimal tree with the sum of branch length = 0.95913905 is shown. The percentage of replicate trees in which the associated taxa clustered together in the bootstrap test (1000 replicates) is shown next to the branches. The tree is drawn to scale, with branch lengths in the same units as those of the evolutionary distances used to infer the phylogenetic tree. The evolutionary distances were computed using the p-distance method and are in the units of the number of base differences per site. All positions containing gaps and missing data were eliminated. *E. coli* ATCC_117775T was used as an out group while LAB producing α-Gal was annotated with pink and blue colored bullets. Evolutionary analyses were conducted using MEGA6.

Isolates were selected for molecular identification based on their ability to produce α-Gal using 16S rRNA gene sequencing. The resulting nucleotides were BLAST searched on National Centre of Biotechnology Information (NCBI; https://www.ncbi.nlm.nih.gov) and EZBioCLoud (https://www.ezbiocloud.net) databases and identified as belonging to *Weissella* (17%) and *Limosilactobacillus* (83%) genera with a percentage similarity of 99–100% (Table 1). The molecular relationship between the isolates is illustrated by a phylogenetic tree (Fig. 2).

**Table 1:** Identity of the selected lactic acid bacteria producing α-Gal.

Isolate	Identified organism	16S rRNA identity (%)	Accession number	References
YZ01	*Limosilactobacillus fermentum*	100	OK083740	This study
KZ06	*Limosilactobacillus fermentum*	100	OK083745	
YA03	*Weissella confusa*	99.64	OK083747	
KA20	*Limosilactobacillus fermentum*	100	MW404623	
KA27	*Limosilactobacillus fermentum*	100	MW404624	
KB30	*Limosilactobacillus fermentum*	100	MW404625	
LBH1148	*Leuconostoc mesenteroides*	ND	NA	^43^
LBH1066	*Lacticaseibacillus paracasei*	99.4	KT000590	^44^
LBH1073	*Levilactobacillus brevis*	99	KT000597	^44^
LBH1074	*Agrilactobacillus composti*	99	KT000598	^44^

NB: Percentage similarity of ≥ 97% to existing 16S rRNA genes in NCBI database was used for organism identification with a minimum of 750 bp region.

### Lactic acid bacteria producing α-Gal exhibited potential probiotic and safety properties

**Figure 3:**
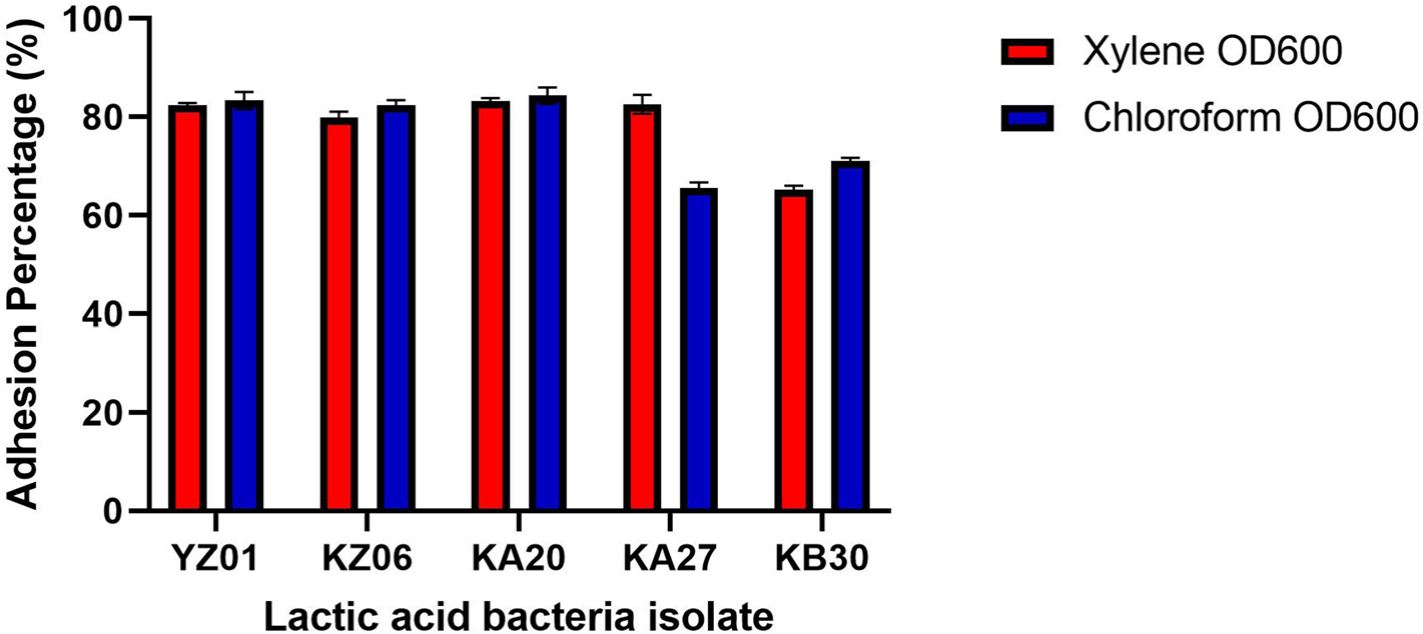
Cell-Surface Hydrophobicity of the lactic acid bacteria. Ability of lactic acid bacteria to adhere to gastrointestinal wall was measured using the microbial adhesion to hydrocarbon method that measures the cell surface hydrophobicity (CSH). The CSH estimated in percentage gives the value of adhesion potential of each bacterial isolates presented in bar chart as a mean of triplicate values. Bars represent the standard deviation from the mean value. *NB:* Cell surface hydrophobicity of Isolates LBH1066, 1073, 1074^44^, LBH1148 (data not available).

The probiotic potential of the α-Gal producing LAB was assessed. Their tolerance to pH 2 varied among the isolates with a growth rate per hour ranging from 5.30 ± 0.02 to 15.24 ± 0.01% with isolate YA03 having the least tolerance to the acidic medium while KZ06 had the highest tolerance (Table 2). Aside *Weissella confusa* YA03, all the other isolates identified as *Limosilactobacillus fermentum* exhibited reasonable viability at pH 2 with OD between 0.8 and 0.9 like observation by Orike et al.^45^. In consideration of their tolerance to bile salt, KB30 had the best tolerability while YA03 had the lowest with a growth rate per hour in medium containing 0.3% bile that ranged from 4.52 ± 0.02 to 13.98 ± 0.01% (Table 2). All the isolates showed no haemolytic property (γ haemolysis) on Colombia agar plates containing 5% (w/v) sheep blood while *E. coli* (β-haemolysis) and *Staphylococcus aureus* (α-haemolysis) served has control for positive haemolysis. Alongside the non-hemolytic property, resistance to Vancomycin and susceptibility to Ampicillin, Cephalothin, Chloramphenicol, Gentamicin, Oxacillin, Erythromycin and Clindamycin showed their phenotypic resistance pattern as an indicator of their safety property as regard to absence of antibiotic resistance (Table 3). Although, isolate KA20 had an intermediate susceptibility to erythromycin, in general none of the selected LAB had an antibiotic resistance profile that is of safety concern. Further, in exploring their ability to adhere to epithelial cells of the gastrointestinal tract which is an important property to determine their propensity to attach to the GIT, their cell surface hydrophobicity (CSH) was studied. All tested strain had > 65% hydrophobicity ability irrespective of the hydrocarbon used with the value ranging from 65.21 ± 0.79—83.12 ± 0.83% in xylene and 65.45 ± 1.22—84.50 ± 1.15% in chloroform (Fig. 3) *L. fermentum* KA20 had the highest CSH which was 83% while *L. fermentum* KB30 had the least with 68% which is consistent with the findings by Harnentis et al.^46^.

**Table 2:** Growth of selected lactic acid bacteria in pH 2, 0.3% bile salt and their hemolytic property.

Isolates	pH 2 (OD_620_)		0.3% Bile (OD_620_)		Hemolysis
	0 h	3 h	0 h	3 h	
YZ01	0.586 ± 0.03^c^	0.905 ± 0.08^c,d^	0.612 ± 0.03^a^	0.839 ± 0.07^a^	No (γ)
KZ06	0.468 ± 0.03^a^	0.925 ± 0.06^d^	0.487 ± 0.03^a^	0.849 ± 0.00^a^	No (γ)
YA03	0.548 ± 0.03^b,c^	0.707 ± 0.01^a^	0.524 ± 0.19^b^	0.659 ± 0.14^b^	No (γ)
KA20	0.496 ± 0.03^a,b^	0.931 ± 0.06^d^	0.505 ± 0.02^a^	0.923 ± 0.01^a^	No (γ)
KA27	0.524 ± 0.04^a,b,c^	0.823 ± 0.01^b,c^	0.517 ± 0.02^a^	0.922 ± 0.03^a^	No (γ)
KB30	0.517 ± 0.04^a,b^	0.798 ± 0.02^b^	0.535 ± 0.03^a^	0.955 ± 0.02^a^	No (γ)

Data represent the mean value of triplicate optical density (OD) taken at 620 nm and expressed as mean ± standard deviation. Values on the same column followed by the same alphabetical superscript are not significantly different from each other at *p* < 0.05 using one way analysis of variance (ANOVA) and Duncan’s multiple range test. Gamma (γ*)* hemolysis represents no hemolysis. NB: Tolerance of Isolates LBH1066, 1073, 1074 to pH and Bile^44^, LBH1148 (Data not available). Hemolytic property of isolate LBH1066, 1073, 1074, 1148 (Data not available).

**Table 3:** Antibiotic susceptibility pattern of lactic acid bacteria strains.

Isolates	AMP, (10 μg)	CEP, (30 μg)	CD, (2 μg)	C, (30 μg)	E, (15 μg)	GEN, (10 μg)	OX, (1 μg)	VA, (30 μg)
YZ01	S	S	S	S	S	R	S	R
KZ06	S	S	S	S	S	I	S	R
KA20	S	S	S	S	I	I	I	R
KA27	S	S	S	S	S	I	I	R
KB30	S	I	I	S	S	I	I	R

*AMP* Ampicillin, *CEP* Cephalothin, *CD* Clindamycin, *C* Chloramphenicol, *E* Erythromycin, *GEN* Gentamicin, *OX* Oxacillin, *VA* Vancomycin, *S* Susceptible, *I* Intermediate, *R* Resistant. NB: Isolates LBH1066, 1073, 1074, 1148 (Data not available).

### Selected lactic acid bacteria did not increase the level of anti α-Gal antibodies in α-Gal deficient mouse

**Figure 4:**
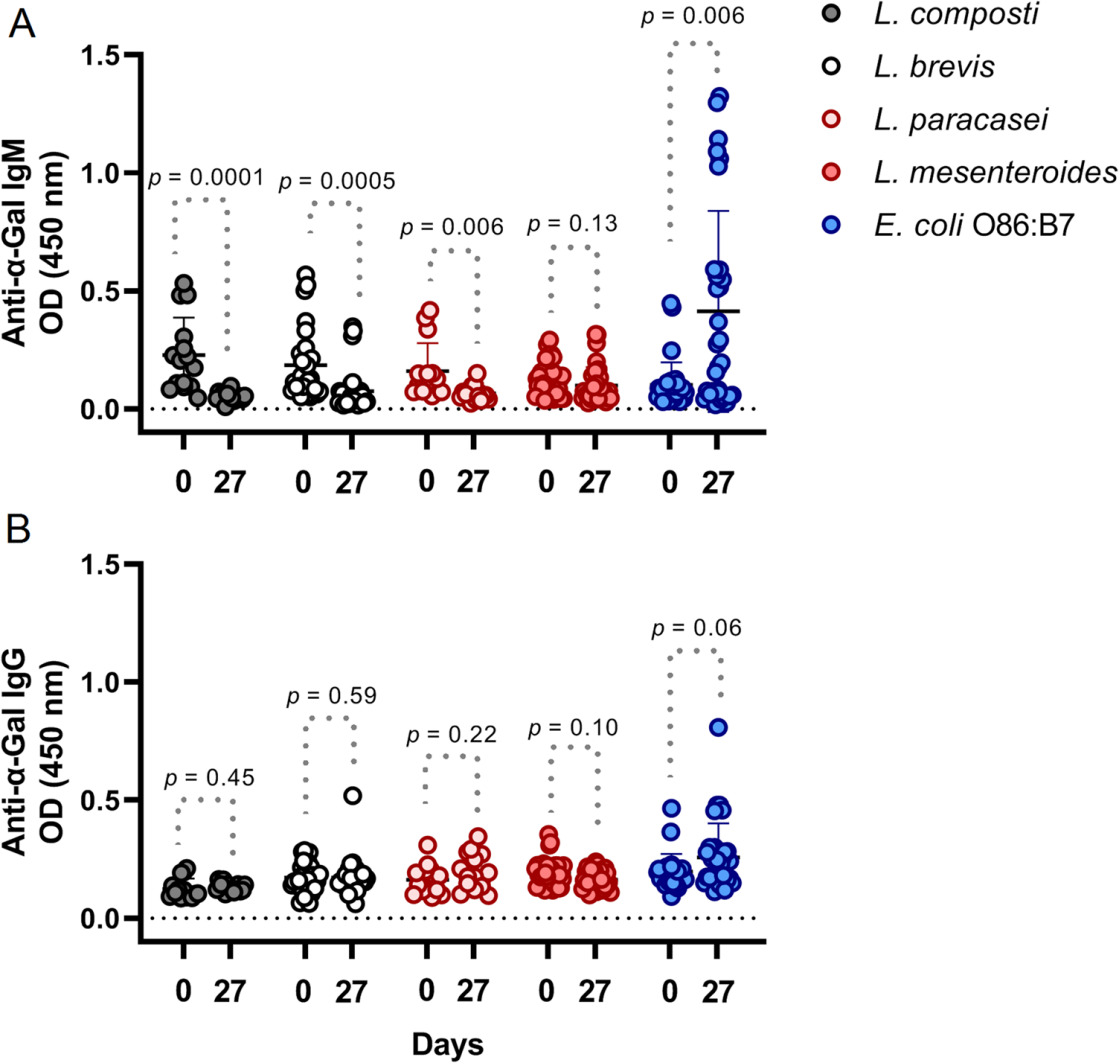
Oral administration of *E. coli* O86:B7 increased anti-Galα1-3Gal IgM antibody response in sera α1,3GT KO mice. Anti Galα1-3Gal IgM levels increased on α1,3GT KO mice more than the measured value in mice treated with *L. brevis* (LBH1073), *L. composti* (LBH1074), *L. paracasei* (LBH1146), and *L. mesenteroides* (LBH1148) by day 27. (**A**) No change was observed on IgG response against Galα1-3Gal antigen. (**B**) The levels of circulating anti-α-Gal IgM and IgG were measured by ELISA. Results shown are means and standard deviation values, IgM was significant at *p* < 0.05, *p* < 0.001, *p* < 0.0001; while IgG was not statistically significant, 2 experiments, n = 30 and three technical replicates per sample.

Previous studies reported that oral administration with bacteria that produce α-Gal in its membrane modified anti α-Gal antibodies response^17,31^. To check the effect of lactic acid bacteria, α1,3GT KO mice were fed with the selected lactic acid bacteria, *L. brevis* (LBH1073), *L. composti* (LBH1074), *L. paracasei* (LBH1066), and *L. mesenteroides* (LBH1148), and a positive control with *E. coli* O86:B7 by oral administration. Sera samples were collected during the experiment and anti-α-Gal antibodies were measured by ELISA. Oral administration with *E. coli* O86:B7 induced an anti-α-Gal IgM response overtime, and a positive tendency to increase on anti-α-Gal IgG was observed. However, anti-α-Gal IgM response observed in mice which received the oral administration of the selected lactic acid bacteria producing α-Gal were much lower at day 27 as compared to *E. coli* O86:B7. The anti-α-Gal IgM titers decreased while an increase was observed with positive control, an indication of an opposite effect of the LAB strains to that of *E. coli* O86:B7. However, there was no effect in IgG across all tested isolates including positive control as the levels of anti-α-Gal IgG were not significant between days 0 and 27 (Fig. 4). Although anti-α-Gal IgE antibodies are not associated with protective response, future studies should include these antibodies as an indicator of possible allergic reactions to α-Gal.

## Discussion

Isolate YA03 was identified as *Weissella confusa* but due to its low tolerance of acidic medium and bile, it was discarded from our experiments. Furthermore, this species has been implicated in opportunistic infections^47^ even though it has been studied and suggested as a probiotic organism due to its natural presence in traditional fermented foods^25,48,49^. The remaining isolates were identified as *L. fermentum, L. brevis, L. composti, L. paracasei*, *Leuconostoc mesenteroides* which according to several studies is a predominant LAB associated with traditional fermented food^26,30^.

One of the key properties considered when selecting a probiotic is its ability to thrive under acidic conditions and be able to tolerate bile salt concentration in the human gut^45,50^. The test for the probiotic potential is extensive, but preliminary investigation in this study reveals that, apart from isolate YA03 which had the lowest tolerance to pH 2 and 0.3% bile, all other isolates were able to tolerate the medium with some level of variability. The variability is expected as the ability to tolerate the harsh condition in the gastrointestinal tract is strain dependent. Considering their growth rate, isolate KZ06 was the most tolerant to acidic medium, while isolate KB30 had better growth in bile medium. Nevertheless, 83% of the α-Gal producing LAB had good tolerance to the acidic and bile medium and were considered to have the probiotic potential for further analysis. This result agrees with the findings of Obioha et al.^26^ who observed no major growth inhibition of *L. fermentum* from dairy product in Enugu for a period of 3 h that it was monitored in acidic medium.

Another desirable property of a probiotic is its ability to colonize and adhere to the intestinal wall such that they are not easily detached during bowel movement. All isolates exhibited hydrophobicity greater than 65% to hydrocarbon. There was no observable statistical difference (*p* < 0.05) in their ability to adhere to either xylene or chloroform. The result obtained in our study is like those reported previously that selected *L. fermentum* as a potential probiotic among all isolates studied for having the highest hydrophobicity^51,52^. The ability to adhere to the mucosal layer is made possible by cell surface proteins and especially mucous adhesion-promoting proteins^52^. In the selection of a probiotic, one of the mandatory properties is the absence of hemolytic activity, a virulence factor common to pathogenic microorganisms. None of the strains showed neither α nor β hemolytic property, a result consistent with previous reports^28,53^.

In addition to safety concerns, LAB should normally be susceptible to erythromycin and clindamycin, but when resistance to erythromycin is observed it has been linked to erythromycin resistance operon—ermB, ermC, ermT, ermG. As for clindamycin, resistance to it has not been genetically understood and whenever it is observed there is usually the presence of erythromycin resistance operon. In many reports, LAB has been shown to be susceptible to erythromycin while they have natural resistance ability to vancomycin^41^. The importance of antibiotic resistance in probiotic selection is to determine if resistance is intrinsic or extrinsic. When resistance to vancomycin is observed, it is intrinsic but when there is resistance to erythromycin or clindamycin it is an acquired resistance and the danger lies in its transferability^54^. In this study aside from isolate KA20 that showed intermediate susceptibility to clindamycin, all other isolates can be considered safe without any potential of carrying transferable antibiotic resistance genes.

The bacterial isolates from this study that are positive for α-Gal production and showed potential probiotic properties need further studies for their possible usage in developing a functional food for immune modulation against some infectious diseases^29,55,56^. Indeed, they can be considered for vaccine development, as carbohydrate-based vaccines are good considerations in control of some pathogens^17^ and can also be considered as a single-antigen pan-vaccine for the control of multiple infectious diseases^57^. Taking into consideration that it is an evolving area of research, a new humanized mouse model has been bred that lacks α-Gal epitopes just like humans with the ability to secrete antibodies against the glycan^58^. This will bridge the gap between the animal model and humans and will be very useful in preclinical investigations. Here, an α1,3GT KO mice were used to investigate the immune response to some selected lactic acid bacteria producing α-Gal. In contrast to the reported anti-α-Gal immune response by α1,3GT KO mice to *E. coli* O86:B7, the anti-α-Gal IgM and IgG level observed were not significant.

Gram-positive bacteria cell wall components do not have highly immunogenic surfaces like Gram-negative bacteria, which might be a reason for the low immune response observed^11–13^. The structure of glycans is also dependent on the source and the primary structure varies between bacteria^59^, and so it should be further explored and compared between LAB and *E. coli*. Another approach could be a prolonged feeding which may change the gut microbiota composition and when α-Gal producing microbes becomes dominant the immune response might be different by raising the levels of circulating α-Gal specific IgM and IgG antibodies during feeding. Engineering a LAB strain to secret lipopolysaccharide specific peptides may be also used to increase the immunogenic surface of α-Gal producing LAB. Even though the genetic manipulation of lactobacilli is very challenging and takes many years before achieving success with strain of interest this study suggests that it is a possibility that could be explored. Likewise, a continuous feeding with the selected α-Gal producing LAB could modify the composition of the gut microbiota and if dominant might modulate the immune response contrary to what was observed in this study. As reported by Mangold et al.^42^, anti-alpha-Gal antibody titers remain unaffected by the consumption of fermented milk containing *Lactobacillus casei* in healthy adults and taxonomically closer probiotics such as *E. coli* Nissle 1917 have been proposed as effective therapies^60^. Nevertheless, the diversity and safety within LAB makes it a good source to explore for promising probiotic strains which this study suggests.

In conclusion, this study is the first attempt to isolate LAB from fermented food for the detection of α-Gal production. The results identified bacteria such as *Limosilactobacillus fermentum, Levilactobacillus brevis, Agrilactobacillus composti, Lacticaseibacillus paracasei and Leuconostoc mesenteroides* containing α-Gal as a potential candidate effective and safe probiotic for the prevention and treatment of infectious diseases. The low immunological response observed from in vivo study could be mitigated by engineering specific strains with modified immunogenic surface, elongated feeding time or continuous search of newer strains with better immune modulation.

## Methods

### Isolation of bacteria from fermented foods

Presumptive LAB was isolated from *kunun-zaki, kindirmo* and *pulque*^44^ a traditionally fermented non-dairy and dairy food. Serial dilution of the fermented food was performed and cultured on de Man, Rogosa Sharpe (MRS) agar (Hi-Media, pH 6.4), a selective medium for LAB and incubated anaerobically at 37 °C for 48 h has previously described (Unpublished data). Colonies were randomly selected based on their morphology and purified by streaking on agar plates to obtain distinct pure colonies that were preserved in MRS broth medium (Hi-Media, pH 6.5) containing 20% (v/v) glycerol at − 20 °C.

### Phenotypic characterization of the isolates

The bacterial isolates were tested for their Gram reaction using a Gram staining kit (Hi-Media, Mumbai), cell morphology was observed at 100 × power microscope (Zeiss, Germany) and catalase reaction using 3% (v/v) hydrogen peroxide was carried out to determine the presence of catalase enzyme. Physiological properties such as growth at 15 °C and 45 °C temperature, different Sodium chloride (NaCl) concentrations (2%, 4% and 6.5% w/v), CO_2_ production from glucose, growth at pH 3, 0.3% bile salt and carbohydrate fermentation profile were studied (unpublished data)^44^.

### Detection of α-Gal

Bacteria were cultured at 37 °C for 18 h in MRS medium (Hi-Media, Mumbai). The bacterial isolates were centrifuged 2 × in PBS (4000 g, 5 min) and re-washed using PBS. Afterwards they were fixed in 4% paraformaldehyde at 25 °C for 30 min before being washed one more time in PBS. The harvested cells after washing were incubated with 3% Human Serum Albumin (I, Sigma-Aldrich) in Phosphate-buffered saline at 25 °C for 1 h. Then, further incubation was done with the α-Gal Epitope (Galα1-3Galβ1-4GlcNAc-R) monoclonal antibody (M86, Enzo Life Sciences, Farmingdale, NY) that have been diluted 1:50 in 3% I/PBS for 14 h at 4 °C. For secondary antibody, FITC-goat anti-mouse IgM (Abcam, Cambridge, UK) onfuse antibody (diluted 1/200 in 3% I/PBS; 1 h at 25 °C) was used. Samples were analyzed on a FAC-Scalibur flow cytometer equipped with CellQuest Pro software (BD Bio-Sciences, Madrid, Spain). The viable cell population was gated according to forward-scatter and side-scatter parameters while bacteria incubated with the secondary antibody only and *E. coli* O86:B7 were used as negative and positive controls respectively according to the method of Cabezas-Cruz et al.^61^ with some modification. Results were presented in geo mean and median of the Fluorescence Intensity (FI) measured.

### Molecular characterization

Genomic DNA of the lactic acid bacteria with α-Gal properties was extracted and 16S rRNA gene were amplified using universal bacteria primer (27F-AGAGTTTGATCCTGGCTCAG and 1429R-GGTTACCTTGTTACGACTT) (GCC Biotech) and further cleaned up using ExoSAP-IT^TM^PCR product clean up reagents (Thermofisher; Cat. No. 78201.1). The PCR amplicon was run on 1% (w/v) agarose gel electrophoresis (Genetix Biotech Asia Pvt. Ltd.), visualized in a gel documentation system (Uvitech, Cambridge) and sequenced by a Sanger sequencing service (Eurofins Genomics India Pvt. Ltd.). The raw data of the sequenced DNA chromatogram were checked using FinchTV. The FASTA file was exported to the MEGA software and aligned using Cluster W package. The obtained 16S rRNA nucleotide sequences of α-Gal producing LAB strain were compared with other reported LAB deposited in NCBI GenBank and BLAST search. A 97–100% similarity to GenBank database was in the same operational taxonomy unit (OUT). The evolutionary analyses were conducted in MEGA 6 by creating a phylogenetic tree of lactic acid bacteria producing α-Gal based on the alignment of the partial 16S rRNA sequences. The evolutionary history was inferred using the Neighbor-Joining method with a bootstrap value of 1000 replicates. The evolutionary distances were computed using the p-distance method and are in the units of the number of base differences per site.

### Examination of probiotic properties of the lactic acid bacteria isolates

#### Tolerance to low pH

Overnight culture of LAB isolates actively growing were harvested by centrifugation (5000 rpm, 10 min, 4 °C) and were washed twice in PBS (pH 7.2). The harvested cells were re-introduced into MRS broth medium lowered to pH 2 using 1 N hydrochloric acid (HCL) followed by incubation at 37 °C under anaerobic condition. Tolerance to low pH was assessed in triplicates by monitoring the cell growth at OD_620_ every hour at 37 °C for 3 h according to the methods used by Orike et al.^45^ with slight modification. MRS broth at pH 6.5 with cell suspension and MRS broth (pH 6.5) with no cell suspension served as positive control and negative control respectively. The percentage growth rate per hour was calculated using the formula Growth rate per hour (%) = [(Final O.D./Initian O.D.) − 1] × 100/TD, where TD is the time difference.

#### Tolerance to 0.3% bile salt

Taking into consideration that the average bile concentration is on average of 0.3% (w/v) in the intestine and the staying time of food in the stomach is projected to be 4 h^62^, the test was carried out using these parameters. Active cultures of LAB were inoculated into MRS medium containing 0.3% bile (Hi-Media) and incubated under anaerobic conditions. Cell growth was monitored every hour and tolerance to 0.3% Bile was assessed in triplicates by taking the OD at 620 nm every hour at 37 °C for 3 h according to Yi et al.^50^ methodology with slight modification. MRS broth at pH 6.5 with cell suspension and MRS broth (pH 6.5) with no cell suspension served as positive control and negative control respectively. The percentage growth rate per hour was calculated using the same formula described above.

#### Hemolytic activity

Overnight culture of LAB isolate was used for the hemolytic activity test in line with the method of Maheshwari et al.^53^. The actively growing LAB isolates were streaked on Columbia blood agar plates containing 5% sheep blood followed by 24 h incubation in an anaerobic jar at 37 °C. Afterwards, the plates were observed for hemolytic features indicated by the colour and clear zones around the bacteria colonies and designated as, γ-non-hemolytic, β and α hemolysis.

#### Antibiotic resistance

Overnight LAB broth culture (100 µl) was pipetted into petri plates in triplicate. Thereafter, 10 ml MRS agar (Hi-Media) was aseptically poured into the petri plate and gently swirled. It was left to solidify before antibiotic discs (Ampicillin (AMP); Cephalothin (CEP); Clindamycin (CD); Chloramphenicol I; Erythromycin I; Gentamicin (GEN); Oxacillin (OX); Vancomycin (VA) were placed on it and kept at room temperature for 30 min to allow the antibiotics to diffuse^63^. The petri dishes were placed in an incubator with 5% CO_2_ for 18 h at 37 °C and the observed zones of inhibition were measured. The zone of inhibition recorded was compared to the standards to determine susceptibility and resistance based on the Minimal Quality Control recommendations for *Streptococcus pneumonia* ATCC 49,619 and *Escherichia coli* ATCC 25,922 used as standard to determine zone of inhibition equivalent to susceptibility. The zone of inhibition between 10–15 mm was considered as intermediate sensitivity, greater than 16 mm is regarded as sensitive while observation of zone of inhibition up to 10 mm was considered as resistant.

#### Microbial adhesion to hydrocarbons

In vitro adhesion was determined by measuring the cell surface hydrophobicity to hydrocarbons using the microbial adhesion to hydrocarbons (MAHC) principle following the methods of Yi et al.^50^ with slight modifications. Overnight incubation of 100 μl of isolates that were introduced into 10 ml of MRS broth was first carried out. The active cultures were centrifuged (8000 rpm, 5 min, 4 °C), re-suspended in PBS (pH 7.4) and washed twice. The cells were further re-suspended in PBS and OD was taken at 600 nm (A_0_). The bacterial cells were mixed with hydrocarbons (Chloroform and Xylene) at the ratio 1:3 (hydrocarbon/bacterial cells) and vortex for 2 min. The resulting mixture was placed in an incubator for 30 min at 37 °C for development of different phases. The liquid phase was pipetted out and OD at 600 nm was taken (A_f_). The fraction partitioned to the hydrocarbon phase is the measure of the adhesion property of the bacteria to hydrocarbons. Thus, the percentage of the cell surface hydrophobicity of the bacteria cells adhering to xylene and chloroform were calculated as Cell Surface Hydrophobicity = [(Ao − Af) × 100].

### In vivo evaluation for anti-α-Gal immunologic response

#### Mice and housing conditions

Six-week-old *ggta1* gene knockout mice (α1,3GT KO mice) were maintained under normal breeding conditions. Animals had permanent access to fresh food and water (normal chow: R 03-40, SAFE), photoperiod of 12-h light cycles, temperature and moisture were carefully controlled in the animal care facilities the Institut national de recherché pour l’agriculture, l’alimentation et l’environnement (IERP, INRAE, Jouy-en-Josas, France). The animals were monitored twice a day (d) by experienced technicians and deviations from normal behaviors or signs of health deterioration were recorded and reported. Experiments were handled in accordance with institutional ethical guidelines, (project 02550.01) and the study was approved by the COMETHEA ethics committee (“Comité d’Ethique en Expérimentation Animale”) of the Centre INRA of Jouy-en-Josas and AgroParisTech. The study is reported in accordance with ARRIVE guidelines (https://arriveguidelines.org).

#### Bacterial cultures and oral administration

The gram-positive bacterium *Levilactobacillus brevis* LBH1073 (CNCM I-5321), *Agrilactobacillus composti* LBH1074, *Lacticaseibacillus paracasei* LBH1146, and *Leuconostoc mesenteroides* LBH1148 (INRAE collection)^43,44^, with highest level of α-Gal were selected and cultured at 37 °C for 18 h in MRS broth (Difco, Bordeaux, France), cells were harvested by centrifugation at 4500 × g for 10 min at 4 °C, rinsed twice with PBS (pH 7.4) and finally re-suspended at 5 × 10^9^ colony-forming units (CFU)/mL in PBS-15% glycerol. After the first serum sampling (d 0), 200 µL containing 1 × 10^9^ CFU of each bacterial strain, or 200 µL of PBS, were administered intragastrically for a period of 3 consecutive days at week 1, week 2 and week 3. The study groups are as follows: PBS (KO-PBS), *L. paracasei* LBH1146 (KO-LBH1146), *L. brevis* LBH1073 (KO-LBH1073), *A. composti* LBH1074 (KO-LBH1074), *L. paracasei* LBH1146 (KO-LBH1146), and *Leuconostoc mesenteroides* LBH1148 (KO-LBH1148).

#### Sera sampling

Mice were bled on days 0th, and 21st. The blood sampling was performed by submandibular bleeding using a lancet according to the Guide to the Ethical Evaluation of Animal Studies, 7.5% circulating mouse blood (~ 100 μl) with a recovery period at least of one week, ensuring the welfare of the mice used. For separation of serum from total blood, a sterile tube without anticoagulant was used. The blood from each mouse was maintained in standing position at room temperature (RT) for clotting (20–30 min) and centrifuged at 2000 × g for 10 min in a refrigerated centrifuge. Serum was collected and conserved at − 80 °C until used for analysis.

#### Determination of anti-α-Gal IgM and IgG antibody levels

Antibodies positive against α-Gal Galα1-3Gal epitope were measured in mice sera using a previously reported protocol^12^. The 96-well ELISA plates (Thermo Scientific, Waltham, MA, USA) were coated with 0.5 µg/m (100 µl/well) of α-Gal linked to BSA (Dextra Laboratories, Reading, UK) diluted in carbonate/bicarbonate buffer (0.05 M, pH 9.6) and incubated for 2 h with 100 rpm shaking at RT. Plates were incubated overnight at 4 °C and then wells were washed three times with 100 µl PBS containing 0.05% (vol/vol) Tween 20 (PBST), and blocked with 1% human serum albumin in PBS (HSA/PBS; 100 µl) for 1 h at RT. After three washes, sera were diluted 1:50 in 0.5% HSA/PBS and incubated for 1 h at 37 °C with 100 rpm shaking. Plates were washed three times and HRP-conjugated goat anti-mice IgG and IgM antibodies (Sigma-Aldrich, St. Louis, MO, USA) were added at 1:1000 dilution in 0.5% HSA/PBS (100 µl/well) and incubated for 1 h at RT with shaking. Plates were then washed three times and the reaction was developed with 100 µl ready-to-use TMB solution (Promega, Madison, WI, USA) at RT for 20 min in the dark. The reaction was stopped with 50 µl of 0.5 M H_2_SO_4_. Optimal antigen concentration and sera dilutions and conjugate were established using a titration assay. The optical density (O.D.) was measured at 450 nm using an ELISA plate reader (Filter-Max F5, Molecular Devices, San Jose, CA, USA). All samples were tested in triplicate and the average value of three blanks (no antibodies) was subtracted from the reads. The cut-off was determined as two times the mean O.D. value of the blank controls.

### Data analysis

The data management and analysis were carried out using Microsoft Excel 2010 and GraphPad Prism v 9.0. The statistical differences of sera reactivity against α-Gal were evaluated using the paired Wilcoxon signed rank test in the GraphPad 9 Prism program (GraphPad Software Inc., San Diego, CA, USA). The comparisons of differences in mean of the tolerance to acidic medium and bile salt were carried out using One-way ANOVA, *p* < 0.05 with the Statistical Package for Social Sciences (SPSS) v.20.

## Data Availability

The data generated during this study are available on reasonable request.

## References

[1] Waldvogel FA (2004). Infectious diseases in the 21st century: Old challenges and new opportunities. Int. J. Infect. Dis..

[2] Pacheco I (2020). Vaccination with alpha-Gal protects against mycobacterial infection in the zebrafish model of tuberculosis. Vaccines.

[3] Crompton PD (2014). Malaria immunity in man and mosquito: Insights into unsolved mysteries of a deadly infectious disease. Annu. Rev. Immunol..

[4] Wikel SK (2018). Ticks and tick-borne infections: Complex ecology, agents, and host interactions. Vet. Sci..

[5] Czaplewski L (2016). Alternatives to antibiotics—A pipeline portfolio review. Lancet. Infect. Dis..

[6] Saraiva RG, Dimopoulos G (2020). Bacterial natural products in the fight against mosquito-transmitted tropical diseases. Nat. Prod. Rep..

[7] Gunasekaran B, Gothandam KM (2020). A review on edible vaccines and their prospects. Braz. J. Med. Biol. Res..

[8] Ngwa CJ, Pradel G (2015). Coming soon: Probiotics-based malaria vaccines. Trends Parasitol..

[9] Hodzic A, Mateos-Hernandez L, de la Fuente J, Cabezas-Cruz A (2020). α-Gal-based vaccines: Advances, opportunities and perspectives. Trends Parasitol..

[10] Hill C (2014). Expert consensus document: The international scientific association for probiotics and prebiotics consensus statement on the scope and appropriate use of the term probiotic. Nat. Rev. Gastroenterol. Hepatol..

[11] Montassier E (2020). Distribution of bacterial α1,3-galactosyltransferase genes in the human gut microbiome. Front. Immunol..

[12] Mateos-Hernández L, Obreg D, Maye J, Borneres J (2020). Anti-tick microbiota vaccine impacts *Ixodes ricinus* performance during feeding. Vaccines.

[13] Cabezas-Cruz A, de la Fuente J (2017). Immunity to α-Gal: The opportunity for malaria and tuberculosis control. Front. Immunol..

[14] Soares MP, Yilmaz B (2016). Microbiota control of malaria transmission. Trends Parasitol..

[15] Galili U (2020). Human natural antibodies to mammalian carbohydrate antigens as unsung heroes protecting against past, present and future viral infections. Antibodies.

[16] Galili U, Anaraki F, Thall A, Hill-black C, Radic M (1993). One percent of human circulating B lympocytes are capable of producing the natural anti-Gal antibody. Blood.

[17] Cabezas-Cruz A, Valdés JJ, de la Fuente J (2016). Control of vector-borne infectious diseases by human immunity against α-Gal. Expert. Rev. Vaccines.

[18] Galili U (2019). Evolution in primates by “Catastrophic-selection” interplay between enveloped virus epidemics, mutated genes of enzymes synthesizing carbohydrate antigens, and natural anti-carbohydrate antibodies. Am. J. Phys. Anthropol..

[19] Cabezas-Cruz A (2015). Regulation of the immune response to α-Gal and vector-borne diseases. Trends Parasitol..

[20] Yilmaz B (2014). Gut microbiota elicits a protective immune response against malaria transmission. Cell.

[21] Pacheco I (2021). Probiotic bacteria with high alpha-Gal content protect zebrafish against mycobacteriosis. Pharmaceuticals.

[22] Dowarah R, Verma AK, Agarwal N, Singh P, Singh BR (2018). Selection and characterization of probiotic lactic acid bacteria and its impact on growth, nutrient digestibility, health and antioxidant status in weaned piglets. PLoS ONE.

[23] Ayivi RD (2020). Lactic acid bacteria: Food safety and human health applications. Dairy.

[24] Zheng J (2020). A taxonomic note on the genus *Lactobacillus*: Description of 23 novel genera, emended description of the genus *Lactobacillus* Beijerinck 1901, and union of Lactobacillaceae and Leuconostocaceae. Int. J. Syst. Evol. Microbiol..

[25] Pasolli E (2020). Large-scale genome-wide analysis links lactic acid bacteria from food with the gut microbiome. Nat. Commun..

[26] Obioha PI (2021). Identification and characterisation of the lactic acid bacteria associated with the traditional fermentation of dairy fermented product. Braz. J. Microbiol..

[27] Koirala S, Anal AK (2021). Probiotics-based foods and beverages as future foods and their overall safety and regulatory claims. Futur. Foods.

[28] Thumu SCR, Halami PM (2020). *In vivo* safety assessment of *Lactobacillus fermentum* strains, evaluation of their cholesterol-lowering ability and intestinal microbial modulation. J. Sci. Food Agric..

[29] Dimidi E, Cox S, Rossi M, Whelan K (2019). Fermented foods: Definitions and characteristics, gastrointestinal health and disease. Nutrients.

[30] Fagbemigun O (2021). Isolation and caracterization of potential starter cultures from the Nigerian fermented milk product nono. Microorganisms.

[31] Mateos-Hernández L (2020). Gut microbiota abrogates anti- α -Gal IgA response in lungs and protects against experimental *Aspergillus* infection in poultry. Vaccines.

[32] Urra JM (2021). The antibody response to the glycan α-Gal correlates with COVID-19 disease symptoms. J. Med. Virol..

[33] Villar M (2021). Characterization by quantitative serum proteomics of immune-related prognostic biomarkers for COVID-19 symptomatology. Front. Immunol..

[34] de la Fuente J, Gortázar C, Cabezas-Cruz A (2021). Immunity to glycan α-Gal and possibilities for the control of COVID-19. Immunotherapy.

[35] Mak JWY, Chan FKL, Ng SC (2020). Probiotics and COVID-19: One size does not fit all. Lancet Gastroenterol. Hepatol..

[36] Taghinezhad-S S (2021). Probiotic-based vaccines may provide effective protection against covid-19 acute respiratory disease. Vaccines.

[37] Breiman A, Ruvën-Clouet N, Le Pendu J (2020). Harnessing the natural anti-glycan immune response to limit the transmission of enveloped viruses such as SARS-CoV-2. PLoS Pathog..

[38] de la Fuente J, de Mera Fernandez IG, Gortázar C (2021). Challenges at the host-arthropod-coronavirus interface and COVID-19: A one health approach. Front. Biosci. (Landmark Ed.).

[39] Sanders ME, Merenstein D, Merrifield CA, Hutkins R (2018). Probiotics for human use. Nutr. Bull..

[40] Kerry GR, Patra JK, Gouda S, Park Y, Shin HS, Das G (2018). Benefaction of probiotics for human health: A review. J. Food Drug Anal..

[41] Nami Y, Bakhshayesh RV, Jalaly HM, Lotfi H, Eslami S, Hejazi MA (2019). Probiotic properties of *enterococcus* isolated from artisanal dairy products. Front. Microbiol..

[42] Mangold A (2012). Anti-alpha-Gal antibody titres remain unaffected by the consumption of fermented milk containing *Lactobacillus casei* in healthy adults. Int. J. Food Sci. Nutr..

[43] Mateos-Hernández L (2021). Anti-microbiota vaccines modulate the tick microbiome in a taxon-specific manner. Front. Immunol..

[44] Torres-Maravilla E (2016). Identification of novel anti-inflammatory probiotic strains isolated from pulque. Appl. Microbiol. Biotechnol..

[45] Orike EL, Adeyemo SM, Omafuvbe BO (2018). Probiotic potentials of lactic acid bacteria isolated from fermenting cassava. Int. J. Probiotics Prebiotics.

[46] Harnentis H (2020). Novel probiotic lactic acid bacteria isolated from indigenous fermented foods from West Sumatera, Indonesia. Vet. World.

[47] Fairfax MR, Lephart PR, Salimnia H (2014). *Weissella confusa*: Problems with identification of an opportunistic pathogen that has been found in fermented foods and proposed as a probiotic. Front. Microbiol..

[48] Franz CMAP (2014). African fermented foods and probiotics. Int. J. Food. Microbiol..

[49] Zimmermann P, Curtis N (2020). Breast milk microbiota: A complex microbiome with multiple impacts and conditioning factors. J. Infect..

[50] Yi R, Tan F, Zhao X (2019). Probiotic properties of *Lactobacillus* strains from traditional fermented yogurt in Xinjiang. E3S Web. Conf..

[51] Tallapragada P, Rayavarapu B, Rao PP, Ranganath NN (2018). Screening of potential probiotic lactic acid bacteria and production of amylase and its partial purification. J. Genet. Eng. Biotechnol..

[52] Rayavarapu B, Tallapragada P, Road M (2019). Evaluation of potential probiotic characters of *Lactobacillus fermentum*. Chem. Chem. Eng. Biotechnol. Food Ind..

[53] Maheshwari SU (2019). Characterization of potential probiotic bacteria from ‘panchamirtham’; A Southern Indian ethinic fermented fruit mix. LWT Food Sci. Technol..

[54] Nubition, D. Comprehensive GRAS assessment of *Lactobacillus plantarum* Lp-115 for usage conditions for general recognition of safety for Danisco USA, Inc (Issue 722). https://www.fda.gov/Food/IngredientsPackagingLabeling/GRAS/NoticeInventory/default.htm%0ADuPont (2017).

[55] Bamgbose T (2021). Functional food for the stimulation of the immune system against malaria. Probiotics Antimicrob..

[56] Chen RY (2021). A microbiota-directed food intervention for undernourished children. N. Engl. J. Med..

[57] Cabezas-Cruz A, de La Fuente J (2017). Immunity to α-Gal: Toward a single-antigen pan-vaccine to control major infectious diseases. ACS Cent. Sci..

[58] Saleh FM (2020). A new humanized mouse model mimics humans in lacking α-Gal epitopes and secreting anti-Gal antibodies. J. Immunol..

[59] Mulloy B, Varki A (2017). Structural Analysis of glycans. Essentials of Glycobiology.

[60] Ukena SN (2007). Probiotic *Escherichia coli* Nissle 1917 inhibits leaky gut by enhancing mucosal integrity. PLoS ONE.

[61] Cabezas-Cruz A (2017). Effect of blood type on anti-a-Gal immunity and the incidence of infectious diseases. Exp. Mol. Med..

[62] Somashekaraiah R, Shruthi B, Deepthi BV, Sreenivasa MY (2019). Probiotic properties of lactic acid bacteria isolated from neera: A naturally fermenting coconut palm nectar. Front. Microbiol..

[63] Shehata AF, Zayed G, Saad OAO, Salwa AHG (2020). Antimicrobial activity and probiotic properties of lactic acid bacteria isolated from traditional fermented dairy products. J. Mod. Res..

